# Supplementary material for: Novel Driver Strength Index highlights important cancer genes in TCGA PanCanAtlas patients
Source: PeerJ. 2022 Aug 11;10:e13860. doi: 10.7717/peerj.13860 (PMC9375969; doi:10.7717/peerj.13860)
Supplement: Supplemental Information 2 [file peerj-10-13860-s002.zip › COHORTS/genes plots/cohorts/2021_8_16_14_9_distribution_cohorts_females_LIHC.pdf]

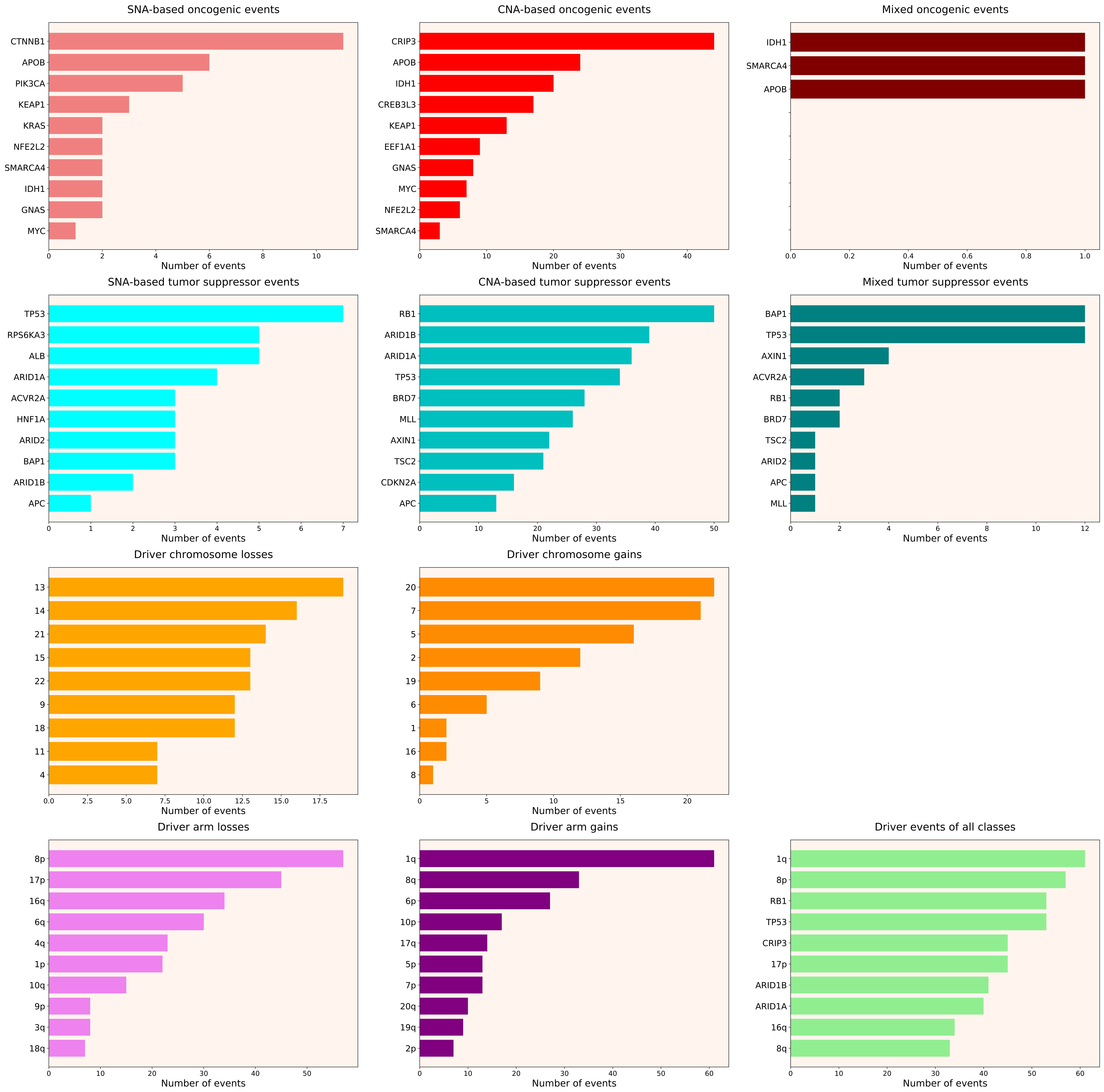
SNA-based oncogenic events
CTNNB1
APOB
PIK3CA
KEAP1
KRAS
NFE2L2
SMARCA4
IDH1
GNAS
MYC
0 2 4 6 8 10
Number of events
CNA-based oncogenic events
CRIP3
APOB
IDH1
CREB3L3
KEAP1
EEF1A1
GNAS
MYC
NFE2L2
SMARCA4
0 10 20 30 40
Number of events
Mixed oncogenic events
IDH1
SMARCA4
APOB
0.0 0.2 0.4 0.6 0.8 1.0
Number of events
SNA-based tumor suppressor events
TP53
RPS6KA3
ALB
ARID1A
ACVR2A
HNF1A
ARID2
BAP1
ARID1B
APC
0 1 2 3 4 5 6 7
Number of events
CNA-based tumor suppressor events
RB1
ARID1B
ARID1A
TP53
BRD7
MLL
AXIN1
TSC2
CDKN2A
APC
0 10 20 30 40 50
Number of events
Mixed tumor suppressor events
BAP1
TP53
AXIN1
ACVR2A
RB1
BRD7
TSC2
ARID2
APC
MLL
0 2 4 6 8 10 12
Number of events
Driver chromosome losses
13
14
21
15
22
9
18
11
4
0.0 2.5 5.0 7.5 10.0 12.5 15.0 17.5
Number of events
Driver chromosome gains
20
7
5
2
19
6
1
16
8
0 5 10 15 20
Number of events
Driver arm losses
8p
17p
16q
6q
4q
1p
10q
9p
3q
18q
0 10 20 30 40 50
Number of events
Driver arm gains
1q
8q
6p
10p
17q
5p
7p
20q
19q
2p
0 10 20 30 40 50 60
Number of events
Driver events of all classes
1q
8p
RB1
TP53
CRIP3
17p
ARID1B
ARID1A
16q
8q
0 10 20 30 40 50 60
Number of events
